# A qualitative study of factors impacting accessing of institutional delivery care in the context of India's cash incentive program

**DOI:** 10.1016/j.socscimed.2017.01.059

**Published:** 2017-04

**Authors:** Sukumar Vellakkal, Hanimi Reddy, Adyya Gupta, Anil Chandran, Jasmine Fledderjohann, David Stuckler

**Affiliations:** a Public Health Foundation of India, New Delhi, India; b Azim Premji University, Bangalore, India; c Save the Children, Gurgaon, India; d University of Adelaide, Adelaide, Australia; e University of Kerala, Trivandrum, India; f Lancaster University, Lancaster, UK; g University of Oxford, Oxford, UK

**Keywords:** India, Janani Suraksha Yojana, Conditional cash transfer, Community health workers, Enabling and impeding factors, Childbirth, Institutional maternal healthcare

## Abstract

Not all eligible women use the available services under India's Janani Suraksha Yojana (JSY), which provides cash incentives to encourage pregnant women to use institutional care for childbirth; limited evidence exists on demand-side factors associated with low program uptake. This study explores the views of women and ASHAs (community health workers) on the use of the JSY and institutional delivery care facilities. In-depth qualitative interviews, carried out in September-November 2013, were completed in the local language by trained interviewers with 112 participants consisting of JSY users/non-users and ASHAs in Jharkhand, Madhya Pradesh and Uttar Pradesh. The interaction of impeding and enabling factors on the use of institutional care for delivery was explored. We found that ASHAs' support services (e.g., arrangement of transport, escort to and support at healthcare facilities) and awareness generation of the benefits of institutional healthcare emerged as major enabling factors. The JSY cash incentive played a lesser role as an enabling factor because of higher opportunity costs in the use of healthcare facilities versus home for childbirth. Trust in the skills of traditional birth-attendants and the notion of childbirth as a ‘natural event’ that requires no healthcare were the most prevalent impeding factors. The belief that a healthcare facility would be needed only in cases of birth complications was also highly prevalent. This often resulted in waiting until the last moments of childbirth to seek institutional healthcare, leading to delay/non-availability of transportation services and inability to reach a delivery facility in time. ASHAs opined that interpersonal communication for awareness generation has a greater influence on use of institutional healthcare, and complementary cash incentives further encourage use. Improving health workers' support services focused on marginalized populations along with better public healthcare facilities are likely to promote the uptake of institutional delivery care in resource-poor settings.

## Highlights

• Cash incentive is not a key enabling factor to accessing institutional delivery care.
• Community health workers' support services is a predominant enabling factor.
• High opportunity costs (monetary and real) is a key impeding factor.
• Skepticism about health service quality and socio-cultural notions are impeding factors.

## 1 Introduction

Although the Millennium Development Goals (MDGs) expired in 2015, India is still far from achieving MDGs 4 (reduce child mortality by two-thirds) and 5 (reduce maternal mortality by three-quarters) (Paul, et al., 2011). As of 2013, there were 52.7 under-five deaths per 1000 live births (Government of India, 2013a) and 167 maternal deaths per 100,000 births (Government of India, 2013b). These deaths were concentrated among lower socioeconomic groups, who report lower levels of accessing of institutional maternal and child healthcare (Pathak et al., 2010, Save The Children, 2010). In 2005 the Government of India launched Janani Suraksha Yojana (JSY), an integral component of National Rural Health Mission (since 2013 the program has been called the National Health Mission [NHM]), to promote the use of maternal and child healthcare facilities. The JSY, launched by modifying the existing National Maternity Benefit Scheme, uses cash incentives to expectant mothers and Accredited Social Health Activists (female community health workers, known as ASHAs) to reduce maternal and neo-natal mortality by promoting institutional delivery. The larger institutional framework of NHM complements the JSY cash incentive by providing comprehensive healthcare, including ante-natal and post-natal services, transport to facilities, and support services from ASHAs. It includes several support services administered by community health workers to encourage pregnant women to use healthcare facilities for childbirth, along with at least three ante-natal check-ups (Government of India, 2014c).

Program implementation was split into 18 high-focus (deprived) and 10 low-focus (developed) states, determined by economic and maternal and child health (MCH) indicators (Government of India, 2014c). In high-focus states, all pregnant women are eligible for the JSY cash incentive of Indian rupee (INR) 1400 (US$1 ≈ INR 68) per birth, and benefits are paid irrespective of birth order, age and socioeconomic position. In low-focus states, the cash incentive is INR 700, and is limited to women below the poverty line, as well as scheduled caste/tribe women (Government of India, 2014a). Those who registered under the JSY but delivered at home are entitled to cash assistance of INR 500 per delivery. In addition, across all states, the JSY provides a transport allowance (INR 300) to eligible expectant mothers who reach healthcare facilities for delivery without the help of ASHA for transport. For expectant mothers reaching facilities with an ASHA's assistance, this amount is provided to the ASHA for arranging the transport (Government of India, 2014c, Government of India, 2015a). The cash incentive is deposited in the bank account of expectant mother. To complement the JSY, the Government of India launched Janani Shishu Suraksha Karyakram (JSSK) on 1st June 2011 with a provision of free transport, diagnostics and drugs to mother and newborn (Government of India, 2015a).

The NHM and its JSY program are implemented in partnership with the state government health departments, with organizational support through officers and health committees at the district, block, and community levels. Every village in the country employs an ASHA to provide support services to women and children. ASHAs are selected from the same village of residence as the expectant mothers. ASHAs are trained to work as an interface between the community and the public health system. They are the first port of call for any health-related demands of deprived sections of the population, especially women and children, who find it difficult to access health services (Government of India, 2014b). ASHAs are entrusted to i) identify pregnant women, ii) facilitate the provision of at least three ante-natal checkups and facility-based childbirth, iii) arrange to immunize the newborn until the age of 14 weeks, iv) make a post-natal visit within 7 days of delivery to track mother's health, v) provide counsel for initiation of breastfeeding to the newborn within one hour of delivery, and continuing for 6 months, and vi) promote family planning (Government of India, 2006). ASHAs are financially incentivized to encourage institutional births (INR 600 per institutional delivery) (Government of India, 2010a, Government of India, 2010b, Government of India, 2014c, Lim et al., 2010, Paul, 2010). From 2009 to 2010 onwards, several state governments revised the JSY guidelines to also promote the provision of ante-natal care. For instance, in 2009, the Chhattisgarh government made ensuring the provision of ante-natal care one of the eligibility conditions for payment of incentives to ASHAs (Government of Chhattisgarh, 2009).

Since its implementation, uptake of the JSY cash incentive has not been very high, particularly in the eight high-focus ‘empowered action group’ states, where of women who gave birth, only 18 percent received the JSY financial benefit in 2007–08 (International Institute for Population Sciences [IIPS] and Macro International, 2010) and 49 percent received the benefit in 2011–12 (Vellakkal et al., 2016). In these states, there was an increase in use of healthcare facilities for childbirth in the post-JSY periods--65 percent of all women who gave birth had used institutional delivery care facilities in the post-JSY period of 2011–12 as compared to 25 percent of all women who gave birth in the pre-JSY period of 2000–04. Yet many pregnant women were not using the available services (Vellakkal et al., 2016). This observation raises an important question: why do many eligible women not use institutional delivery care facilities to receive the JSY cash incentive?

Extant research has pointed to some possible explanations. Low levels of information communication and education activities with emphasis on MCH is associated with lower utilization of the JSY, especially in rural areas (Sharma et al., 2011). In a rural area of Varanasi of Uttar Pradesh, lower level of awareness about the JSY was reported among women of reproductive age group, with a significant negative effect of factors like literacy status of pregnant women and their spouses (Kaushik et al., 2010). In the Ujjain district of Madhya Pradesh, the non-availability of transportation, and maternal perceptions that previous non-institutional deliveries were ‘easy’ were also associated with the non-use of the JSY (Sidney et al., 2012). In the state of Jharkhand, although some women were willing to opt for institutional delivery, several obstacles prevented uptake of the JSY cash incentive and institutional delivery care, such as poor infrastructure, poor quality of care, difficulties in accessing cash incentive, and corruption in disbursement of incentives (Rai et al., 2011). Varied perception of eligibility guidelines in different states, awareness of the program, the amount disbursed, documentation, delays in disbursement to beneficiaries and low or irregular financial incentives to ASHAs were reported as the operation barriers of the JSY (Zodpey and VK, 2014). A study in the state of Madhya Pradesh reported environmental factors that impeded skilled birth attendance, including a chaotic delivery environment, lack of staff preparedness, and unfriendly behavior of staff towards patients (Chaturvedi et al., 2015). Regional imbalances in the quality of the health infrastructure also endure, with high-focus states lagging behind in implementation as compared to low-focus states (Bhattacharyya et al., 2012, United Nations Population Fund- India, 2009).

On the other hand, a recent large-scale household survey revealed that 59 percent of the women from high-focus states were aware of the JSY (UNICEF, 2009). Awareness of the JSY was higher among women from below the poverty line families (64 percent) than above the poverty line families (58 percent), and scheduled caste and scheduled tribe women had higher awareness of the program than women of general caste (UNICEF, 2009). An evaluation study in the states of Uttar Pradesh, Madhya Pradesh, Jharkhand, Orissa, Assam, Jammu & Kashmir, and Tamilnadu showed that since the inception of National Rural Health Mission there was marked improvement of basic health infrastructure with adequate supply of human resource, material, drugs, equipment, and transport system (Planning Commission, 2011).

Against this backdrop, this paper explores the factors impacting the accessing of institutional delivery care facilities in the context of the JSY cash incentive and the NHM. We use qualitative in-depth interviews in the three JSY high-focus Indian states: Jharkhand, Madhya Pradesh and Uttar Pradesh.

## 2 Materials and methods

### 2.1 Study design

This is a cross-sectional qualitative study. Individual face-to-face in-depth interviews (IDI) were conducted.

### 2.2 Study settings

We collected data for this study in the high-focus states of Jharkhand (lower JSY uptake, 24 percent in 2012–13), Madhya Pradesh (higher JSY uptake, 73 percent in 2012–13) and Uttar Pradesh (medium JSY uptake, 36 percent in 2012–13) (Office of the Registrar General and Census Commissioner, 2016). A summary of the socioeconomic and health indicators of the 3 states is given in Table 1.

### 2.3 Participants

With the goal of assessing the factors impacting the accessing of an institutional delivery care facility from the users' perspectives, we interviewed eligible women (n = 41), defined as women who gave birth to a child anytime within the past six months to one year prior to the date of interview, their spouses (n = 44), and their co-residential mothers-in-law (n = 11). However, only one participant was selected from each household--either the eligible woman or her spouse or mother-in-law--so that we could collect a diversity of insights from more families given the limited resources available for the data collection. The spouses and mothers-in-law of eligible women were included in the study because of their dominant role in the decision-making in the Indian family context. Both women who received and did not receive the JSY cash incentive and those who delivered at an institutional delivery care facility and at home were included in the sampling frame. The JSY users who opted for home delivery are defined as those women who gave birth to a child at home but received the JSY cash incentive. We aimed to collect data from roughly an equal number of the JSY users versus non-users, and also an equal number from women with institutional versus home deliveries. In addition, several ASHAs (n = 12) were interviewed in order to provide complementary perspectives on the factors impacting the accessing of institutional delivery care facilities, as they frequently interact with the people at the local level.

### 2.4 Sample selection and data collection

We used a stratified purposive sampling strategy to identify eligible participants. Of the two districts selected from within each state, one was relatively more developed (i.e., with improved infrastructure and more urban and semi-urban features) while the other one was less developed (i.e., with less infrastructure and the presence of large number of remote rural areas). Two blocks from each district, based on their level of development (as with the states), were further selected. Two villages from each block were randomly selected as the final sampling unit for identifying eligible women, spouses and mothers-in-law. To avoid potential selection bias, we refrained from identifying the participants (eligible women, spouses and mothers-in-law) through the JSY and healthcare facility related stakeholders, such as ASHAs, health workers and the JSY officials. Instead, participants were identified after developing rapport with some villagers whom we randomly met at various locations. Importantly, the research team and interviewers consulted each other on the final identification of the participants to ensure that the pool of the selected participants was a mix of those who did and did not receive the JSY incentive, and those who delivered at home versus in a facility (as outlined above). Efforts were made to include participants residing in remote locations. One ASHA was randomly selected from each block. Of the total number invited to interview, 64 percent of the users/non-users of the JSY and 86 percent of the ASHAs agreed to participate.

Interview guides were prepared in the local language (Hindi). Participants were interviewed by trained interviewers at their residences. Probes were used as needed wherever extraction of more relevant information was possible. The interviews were conducted in Hindi. Interviews lasted for between 25 and 50 min. All interviews were tape-recorded and then back-translated into English to maintain accuracy. Interviews were transcribed verbatim by the interviewer. The transcribed interviews were further cross-checked with the recordings by the researchers to ascertain quality and validity. Notes were also taken during the interview to ensure no relevant information was omitted. Summary sheets were used to capture the socio-demographic details of the participants.

The interviewers included both males and females; most of them had educational qualifications of bachelor's degrees and above, and all of them had previous experience conducting qualitative interviews. They were given further training by the study team both on the basics of the JSY and on their interviewing skills. Prior to the final interviews, a pre-test was conducted among the interviewers to check the appropriateness of the interview guide, to respond to queries raised by the interviewers, and resolve any differences that emerged during the process. The researchers closely monitored the data collection procedures by physically accompanying the interview team. The study was carried out over a period of 3 months from September to November 2013.

### 2.5 Data analysis

Data analysis was carried out using the basic underlying principle of grounded thematic framework theory (Corbin and Strauss, 2008, Strauss and Corbin, 1998). The transcripts were color coded using Atlas-Ti software. Following grounded theory, the transcripts were read and re-read thoroughly by the authors (SV and AG) and theories that repeatedly emerged were highlighted. To identify repeated themes and gain additional insights on the variations in responses between persons and locations, the transcripts were grouped by type of participants and districts for the reading. The themes generated were revised after a series of discussions between authors to avoid personal bias, and then discussed with other co-authors.

We initially followed the inductive approach to derive the themes, followed by the deductive approach using the conceptual framework of health service use outlined by Andersen (1995). Andersen's model argues that healthcare utilization is a function of:

• Environment (i. healthcare system characteristics, e.g., policy, resources, organization, and ii. external environment)
• Population characteristics (i. predisposition characteristics such as demographic, social structure, and health beliefs, ii. resources such as personal, family and community level resources, and iii. realization of the need for care)
• Health behavior (i. personal health practices and ii. use of health services),
• Outcomes (i. perceived health status, ii. evaluated health status, and iii. consumer satisfaction)

For the assurance of analytic rigor, the senior co-authors reviewed the qualitative data analysis (JF) and the presentation of themes and discussion (DS, HR, and AC). The detailed discussions on the themes among the co-authors were also helpful to minimize the researchers' own position on the research findings. Themes were corroborated with secondary data and available literature for better triangulation. A comprehensive network of pathways of utilization and non-utilization of services was then developed. Quotations which were illustrative of the thematic material were selected for presentation.

The study protocol was reviewed and approved by the Institutional Ethics Committee, Public Health Foundation of India, New Delhi. Participation was voluntary, and oral informed consent (recorded) was obtained from all participants before the interview. Participants were informed that the interviews were implemented for research purpose, that their identity will not be disclosed for any reason, and the study findings may be incorporated in to policy to improve institutional delivery care services. Female participants (except ASHAs) were interviewed by female interviewers only. Other than the interviewer and participant, no other people, including family members, were present in the interview site. No incentives, either cash or in-kind, were provided to the participants.

## 3 Results

### 3.1 Participants' characteristics

A total of 108 (36 JSY users, 60 non-users, and 12 ASHAs) interviews were conducted (Table 2). The participants' age ranged from 18 to 40 years for the JSY users/non-users, and 20 years–40 years for ASHAs. Most of the eligible women were housewives and few were laborers. Most JSY users and non-users were illiterate or had attained primary level education, whereas ASHAs had, on average, acquired education up to secondary and higher secondary level, with a few being college graduates as well.

### 3.2 Awareness of the JSY

Most of the participants were aware of the JSY, explaining it as a program where the government provides money for an institutional delivery, free ambulance services for childbirth, and free medicine and food at government hospitals. The JSY users were more familiar with specific details of the program than non-users: while non-users were aware of the JSY, fewer of these participants knew either the name of the program or the amount of the cash transfer. Most participants cited ASHAs as their major source of information on the JSY. Less common but also cited as sources of information on the JSY were the radio, TV, relatives, neighbors, Anganwadi workers (who provide non-formal pre-school education, supplementary nutrition, and nutrition and health education through the government's Integrated Child Development Services program), and hospitals.

(Money is given after delivery, [and a] ‘mamata vahan’ [maternity van/ambulance] is provided to drop to hospital. [Mother: institutional delivery, JSY user])

(I heard some money is given for delivery at government hospital. [Mother: home delivery, JSY non-user])

### 3.3 Factors impacting the accessing of institutional delivery

**Fig. 1:**
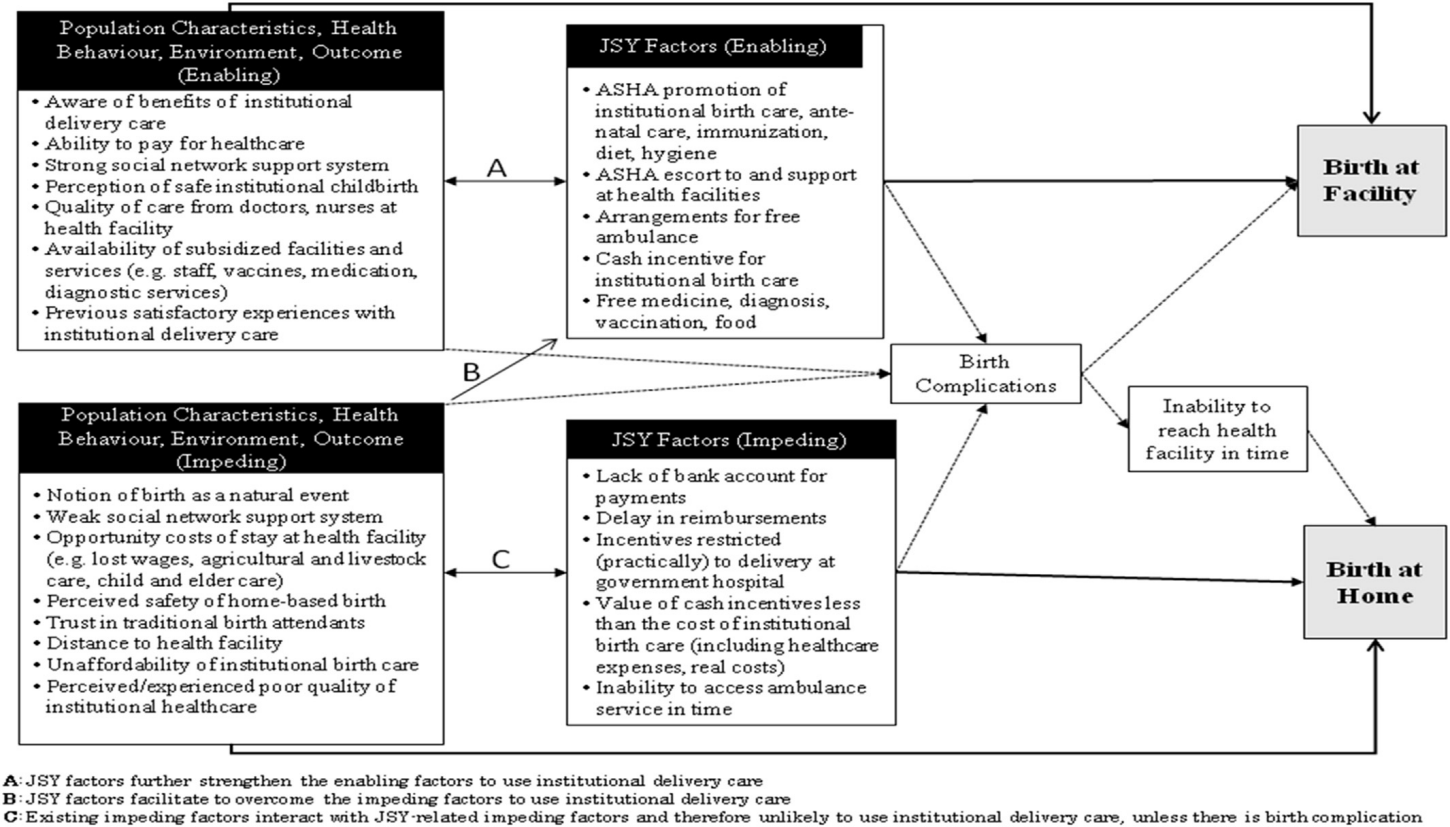
Major enabling and impeding factors and the pathways in accessing of institutional birth care in the context of India's JSY and NHM.

Our analysis showed that the use of an institutional delivery care facility is determined by several impeding and enabling factors, falling into three broad categories: 1) Population characteristic-related factors, 2) Health system and environmental-related factors, and 3) JSY-related factors. Fig. 1 shows the pathways to the use of an institutional delivery care facility, focusing on major factors. First, the population characteristics and health system-related factors affect decision-making on whether or not to seek an institutional delivery care facility. Subsequently, the JSY-related factors such as i) ASHAs' support services, ii) other specific and additional health services offered under the JSY, and iii) the cash incentive interact with the socioeconomic and health system-related factors to further influence the decision and incidence of seeking an institutional delivery care facility.

#### 3.3.1 Population characteristic-related factors

The most commonly reported enabling factor in the decision to use an institutional delivery care facility is the population characteristics, such as the awareness among pregnant women and family members about the value of institutional delivery care facilities for safe childbirth.

(It is better to go hospital for the safety of mother and new born … at home we cannot manage birth complications. [Spouse: institutional delivery, JSY user])

The ability to pay for healthcare also positively affects the decision to use an institutional delivery care facility. This result is based on the opinion of ASHAS and our analysis of socioeconomic status desire to use institutional delivery care rather than on direct quotes from the interviews with JSY users/non-users.

(Many women are from poor economic backgrounds, mostly daily workers. Using hospital for child birth is unaffordable for them due to expenses on medicines and ‘side-payments’ [bribe] to hospital staff [ASHA, Ghaziabad, Uttar Pradesh])

On the other hand, reported impeding factors include socio-cultural elements, reflecting the lack awareness on the need of institutional delivery care facility, and the notion of birth as a natural event needing no institutional delivery care.

(In fact, I have no need to go to hospital … When I did not face [a] problem then why should I go for Diagnosis/Test. [Mother: home delivery, JSY non-user])

(It is my family practice to give birth at home. It is safe. I am comfortable at home. [Mother: home delivery, JSY non-user])

(My all 3 children were delivered at home. Nothing wrong had happened to me and my children. [Mother: home delivery, JSY non-user])

(We didn't need to go anywhere (hospital) and if God is giving everything without any difficulty so what's the problem. [Mother: home delivery, JSY non-user])

We also found that parity played a major role in accessing of an institutional delivery care facility. Primiparous women, as compared to multiparous women (multiparous women in their 30s in particular), expressed preference for an institutional delivery care facility. In our sample, approximately 78 percent of primiparous women and 46 percent of multiparous women had used an institutional delivery facility. The decision of whether and when to seek obstetric care was not taken exclusively by one person in the family. Rather, decision-making appeared to be a household process which evolved across the course of pregnancy. In most cases where obstetric care was sought, we found that the expectant mothers showed more interest in seeking care at a facility, and spouses and other family members often supported. Overall, the decision to seek delivery care evolved over time, and was substantially influenced by the enabling and impeding factors outlined in this study.

#### 3.3.2 Health system and environmental-related factors

The health behavior of the population is impacted by previous interactions with healthcare settings. This may include not only women's own previous experiences directly related to delivery care (e.g., during ante-natal care checkups), but also experiences with non-obstetric services by the expectant mother, as well as healthcare experiences of other family members. Likewise, perceived and/or experienced safe childbirth at institutional delivery care facilities also acts as an additional enabling factor. Participants also commonly highlighted the availability of good quality institutional delivery care and support from nurses and doctors, and fully or partially subsidized institutional delivery care facilities including doctor and nurse services, medicines and diagnostic services as enabling factors.

(At hospitals, there is security of health. Doctors and nurses facilities are available there. [Mother: institutional delivery, JSY user])

Health environment factors such as distance to an institutional delivery care facility and inability to afford healthcare were commonly cited by participants as impeding factors. Distance to an institutional delivery care facility, unaffordable institutional delivery care, and the poor quality of care (perceived and/or experienced), particularly in government institutional delivery care facilities, factored into decision-making as impeding factors.

(Mostly the nurses, not doctors, handle the delivery care in government hospitals [Spouse: home delivery, JSY non-user])

(In hospitals, good doctors are not there. Electricity and water is not always available [Mother: institutional delivery, JSY user]).

Previous experiences of safe childbirth at home and trust in traditional birth attendants (called dai) are other impeding factors. Most multiparous women who had safe births at hospital continued to opt for an institutional delivery care facility. However, there were a few instances of older women opting for home birth even if they had previous birth at facilities. This was mainly influenced by the unsatisfactory quality of services available at facilities, and the perception that the delivery can be made without the support of medical personnel at home among those who had a previous normal vaginal delivery at an institutional delivery care facility.

(The dai has several years of experience. She is taking care of all the delivery in our village. She knows everything. She helps in delivery without much pain by putting oil. [Mother-in-law: home delivery, JSY non-user])

In most instances, we found that the preferences for home for childbirth over institutional delivery care facility were highly prevalent, because of either previous episodes of or the perceived possibility of a safe childbirth at home, and the notion of birth as a natural event needing no institutional delivery care. This resulted in a moderate preference among some women and their families for using an institutional delivery care facility. Thus, an institutional delivery care facility will be opted for on the basis of any anticipated or emergent complications in childbirth. Most participants who experienced an emergent complication did not get admitted to an institutional delivery care facility well in advance, resulting in waiting until the last moment of labor-pain at home before proceeding to an institutional delivery care facility. This finally resulted, in most cases, in the inability to reach to an institutional delivery care facility on time in such short span of time due to distance to institutional delivery care facilities and the non-availability of transport facilities on short notice.

([We] will go to hospital if any complication comes up. [Mother: home delivery, JSY non-user])

(I wanted to go hospital. Mausi [Aunty] was saying ‘Let's go to the hospital’. But I didn't get the chance, as this child was delivered very easily. [Mother: home delivery, JSY non-user])

([The] vehicle [ambulance] was unable to reach [the house in time], so [my child was] born at home; [a] phone call was made to ‘sister-ji’ [ASHA], [the] vehicle did not come, and [my] child was delivered at home. [Mother: home delivery, JSY non-user])

[Sometimes the van (ambulance) facilities to hospital are available and sometimes not. (Mother: home delivery, JSY non-user)]

In addition, lack of a caretaker at home for other small children of expectant mothers was also reported to impede accessing of institutional delivery care.

([We have] no vehicle at home. I was going to hospital and child was born on the way, then I thought I will not go to hospital because it gives lots of trouble. And I have small children at home, who will take care of them? [Mother: home delivery, JSY non-user])

#### 3.3.3 JSY-related factors

Several JSY-related factors served as important enabling factors for several pregnant women and their families on switching their preferences from home to institutional delivery care facilities. We found ASHAs' support services and the cash incentive as two often-mentioned JSY-related factors.

##### 3.3.3.1 ASHAs' support services

The ASHAs played crucial roles in countering several impeding factors and strengthening enabling factors through awareness generation and provision of support services. ASHAs provide person-centered awareness (i.e., interpersonal communication for awareness generation) to pregnant women and their family members on the necessity of childbirth at institutional delivery care settings, ante-natal care, healthy diets, and hygiene for the better health of mother and child. ASHAs invest considerable efforts in identifying pregnant women and building trust with expectant mothers and their family members. Since ASHAs are from the same village of the expectant mothers and are thus familiar with them and their family circumstances, the identification of each expectant mother is done either through direct observations or through speaking to relatives and neighbors. This is followed by building rapport with the woman and her family members (especially with the mothers-in-law, and other senior female members), and registering them under the JSY. A minimum of three counseling sessions for each expectant mother are conducted, occurring every alternate month during the ante-natal phases. This process helps to establish a relationship of trust between ASHAs and expectant mothers (and their families), which our findings suggest may aid ASHAs in overcoming impeding factors.

(We visit village and every house of the village, and ask people about newly married women or about women who are expecting. And if we get to know something like this then we register them under the JSY and counsel them. [ASHA: Hamirpur district, Uttar Pradesh])

(We counsel about the importance of regular ante-natal check-ups for safe delivery and health of newborn, and also about cleanliness, washing of hands with soap after toilet, and also about feeding the child with clean hand. [ASHA: Gwalior district, Madhya Pradesh])

Additionally, ASHAs provide several support services to pregnant women such as: detecting pregnancy using pregnancy kits, registering for ante-natal care at an institutional delivery care facility, ensuring that at least three ante-natal check-ups and all necessary vaccinations are provided to her, making periodic home visits (for providing basic ante-natal support to identify and refer for any potential risks and complications, and for counseling expectant mother and family), arranging of ambulance services, escorting to institutional delivery care facilities, facilitating communication with institutional delivery care providers, and in post-natal care including arranging necessary vaccinations for the newborn.

(This was my first delivery and Sahiya [ASHA] suggested for the hospital, so only due to this we went hospital. [Mother: institutional delivery, JSY user])

(ASHA said take her [pregnant woman] to the hospitals, and the people of my village also said to take to hospital. [Mother-in-law: institutional delivery, JSY user])

(She [ASHA] helped us from beginning to end. She was with us while going to hospitals and also in the hospitals, calling doctors, helping in tests/check-ups. [Spouse: institutional delivery, JSY user])

##### 3.3.3.2 Cash incentive and opportunity costs

Importantly, we found that the cash incentive played a lesser role in motivating the people to opt for institutional delivery care because of the higher associated opportunity costs. In most cases, it was reported that the net value of the JSY cash incentive is less than the total expenses of facility-based childbirth, in terms of both monetary and real costs. Specific monetary costs included healthcare expenditure, food and transport expenses for the mother and her caregivers, and loss of wages for spouses. The real cost component consisted of discomfort of staying at an institutional delivery care facility (and, relatedly, the sacrifice of the comfort of staying at home with family members), inability to attend the regular needs of children and elderly family members, loss of schooling for children, interruptions in spouses' employment participation, and neglect of livestock and agriculture. Moreover, the cash incentive transfer-related factors such as requirement of a bank account for receiving payments, delay in reimbursement, and limitation of the cash incentive to delivery at government and accredited private institutional delivery care facilities also made the JSY less attractive to potential users.

(I didn't want to waste my four days. I didn't want to waste my four days wages. What is advantage there only INR 1000 or 1200. I didn't have a bank account. For bank account INR 1000 will go. Need more money in hospital. Here just giving INR 500 to dai is okay. What we finally get is nothing. [Spouse: home delivery, JSY non-user])

(When we stay at [the] hospital for 3–4 days, who will take care [of] our children and parents, who will take care of agriculture and animals? [Spouse: home delivery, JSY non-user])

(Delivery at hospitals needs money. We need to buy medicines. So many other payments to doctors, nurses and attendants [as side payments], we need to buy special food for mother at hospital [Spouse: institutional delivery, JSY non-user])

(We don't know how long we need to stay at hospital. Mine is not a government job, and if I don't go to my daily job, they will find another person to replace me. [Spouse: home delivery, JSY non-user])

### 3.4 ASHAs' views on enabling and impeding factors

Overall, ASHAs noted that awareness generation about the benefits of institutional delivery care has considerable influence among people to encourage use institutional delivery care facilities. Further, if the pregnant women and families are convinced of the importance of using institutional delivery care facilities, then the JSY cash-transfer incentivizes them further.

(JSY financial incentive does not attract people much, they are attracted to going hospital when we give more information on what all the facilities available there, the free services, and the problems they would face at home deliveries and also on the dai … people consider the safe childbirth is more important than money. [ASHA: Tikamgarh district, Madhya Pradesh])

In some cases, however, the prevailing socio-cultural norms impede the efforts of ASHAs.

(But most people don't fully listen to us; they have their own strong views on whether or not to use institutional delivery care facilities for delivery. [ASHA: Ranchi district, Jharkhand])

According to the ASHAs, the lack of sufficient infrastructure, poor quality of care and several related expenses at government hospitals also constraint people to use the JSY cash incentive and institutional delivery care.

(Not many facilities are often available in government hospitals … they raise lots of concerns of the quality and often tell us that nurses and doctors will demand money in government hospital … sometime staffs won't be there on holidays and weekends, and night ….for caesarian they refer to private hospitals … and sometime it is difficult to get the ambulance, particularly in the night. [ASHA: Chatra district, Jharkhand])

However, the JSY cash incentive component to pregnant women also serves as one of the windows of opportunity for ASHAs for approaching the pregnant women and their families for promoting the use of institutional delivery care facilities.

(I tell them [pregnant women and their families] that at hospitals you will get good care, food and medicine. On top, you will get some money from government as well. [ASHA: Ghaziabad district, Uttar Pradesh])

ASHAs also mentioned that they received only a small monetary reward from the government, and delays in reimbursement of monetary benefits. The poor pay and delays in payment make them lose interest in their work, leading to lack of motivation to encourage institutional deliveries.

### 3.5 Between-state and between-participant variation

We also found slight variation in the factors impacting the accessing of institutional delivery care facilities across the three states. The weak preference for an institutional delivery care facility was mostly found among the participants in Jharkhand. In Jharkhand, the impeding factors such as the trust in the skills of traditional health attendants, the notion of childbirth as a ‘natural event’ to take place at home, the distance to an institutional delivery care facility, and perceived poor quality of institutional delivery care facilities were highly prevalent. To some extent, similar patterns were observed in the state of Uttar Pradesh. In Madhya Pradesh, we found that the participants cited their previous bad experiences with the institutional delivery and other government institutional delivery care facilities, as well as quality-related issues including corruption (bribes) and lack of doctors and nurses--particularly on holidays and during nights--as some of their challenges. It was also reported that often government hospitals made referrals to private hospitals for treatment for birth complications, including those requiring caesarean deliveries. Regarding variation by participant type, no substantial variations were observed between eligible women and mothers-in-law; however, most spouses of the pregnant women cited high opportunity costs (including both monetary and real costs) of using a institutional delivery care facility as major impeding factors.

## 4 Discussion

### 4.1 Key findings

This study explored views on the use of the JSY cash incentive and institutional delivery care facilities across the 3 large high-focus JSY states. We identified a number of enabling and impeding factors that strongly shaped decisions of women and families to opt into or out of the JSY and institutional delivery care for childbirth. The factors fell into three broad categories: population characteristics-related, healthcare system and environmental-related and JSY-related factors. ASHAs' support services to pregnant women, including arrangement of transport facilities, escort to and support at institutional delivery care facilities, and provision of information about the benefits of using an institutional delivery care facility, emerged as the most salient enabling factors. The health workers' consistent support and services along with the improved institutional delivery care services were most instrumental in switching the preferences of people from ‘home’ to a health-facility as place for childbirth. In contrast, the cash incentive component of the JSY was less influential in serving as an enabling factor to access institutional delivery care facility. This was due to the higher opportunity costs (monetary and real costs) associated with use of health facility over home for childbirth, as well as to the inability of the JSY payments to fully cover the monetary costs of institutional delivery, particularly for disadvantaged groups who may lack access to infrastructure (e.g., bank accounts, private vehicles). Our study also highlighted importance of parity in accessing of an institutional delivery care facility.

### 4.2 Relevance of the study findings with the literature and policy

Several quantitative studies have found that the JSY cash incentive was associated with increases in facility-based births (Randive et al., 2013) and declines in neonatal mortality (Gupta et al., 2012, Lim et al., 2010, Panja et al., 2012), improvement in immunization rates and breastfeeding practices (Carvalho et al., 2014), declines in socioeconomic inequality in access to facility-based care in poorer states, and a decline in maternal mortality (Randive et al., 2014). Those studies exclusively focused on the cash incentive elements, without examining the role played by other components that complement the JSY, especially the ASHA support services and additional health infrastructure of NHM. This study provided an in-depth qualitative investigation of the role of various factors impacting the accessing of institutional delivery care facilities in the contexts of the JSY. The cash incentive alone is insufficient to overcome impeding factors such as socio-cultural notions about the perceived safety of home deliveries and health system-related factors. Moreover, the cash incentive is insufficient to overcome the monetary and real costs of an institutional delivery. Higher opportunity costs (in monetary and real terms) in opting for institutional delivery care facilities for childbirth were identified in our study; however simply increasing the amount of cash incentives alone would be insufficient to overcome barriers because utilization of institutional delivery care is influenced by several inter-related socioeconomic, cultural and health system factors. If these factors can be addressed through increased awareness of the benefits of using institutional delivery care through well-equipped healthcare facilities, then the cash incentive can serve as a supplementary incentive.

We also found that there is a socio-cultural preference for home-based childbirth over the facility-based childbirth. This kind of preference for home-based childbirth was also identified in upper-middle income countries like Mexico (Hunt et al., 2002). Previous studies have reported that the quality of care is an important factor impacting maternal care. One study reported that improved public institutional delivery care facilities and skilled female health workers significantly encouraged deliveries at public institutional delivery care facilities in India (Patel and Ladusingh, 2015). As highlighted by one study, there is a need to ensure quality obstetric care prior to increasing coverage of institutional facility-based births if cash transfer programs like the JSY are to improve health outcomes (Chaturvedi et al., 2015). Our findings highlight the importance of community health workers in the Indian healthcare system in order to improve accessing of healthcare-- not only for delivery care but also for promoting other health services such as infant and child care and family planning. As of December 2014, 859,331 ASHAs were in place (Government of India, 2015b). We found that ASHAs were responsible for providing most of the basic necessary support services, such as 1) identifying pregnant women in their communities; 2) providing expectant mothers with counsel on MCH issues, birth preparedness, importance of safe delivery, breastfeeding and complementary feeding, immunization, and contraception; and 3) assisting them in seeking maternal health services, including arranging transport and accompanying them to institutional delivery care facilities. While some observers have raised the possibility that the incentivization of ASHAs to encourage institutional births may discourage provision of non-incentivized services, no study has empirically examined this concern. Future research is needed to examine how incentives to ASHAs may shape the provision of counseling and services.

ASHAs' support serves as a bridge between expectant mothers and institutional delivery care facility stakeholders. This has considerable implications for the expectant mothers and their families, who are encouraged by ASHAs to overcome the discomfort in interacting with the institutional delivery care facilities. This may be particularly true for people from lower socioeconomic backgrounds, who are unfamiliar with the setting of institutional delivery care facilities. Previous work from a large household level survey found that in high-focus states, ASHAs had accompanied 54 percent and stayed with 49 percent of all the women who gave birth in government institutional delivery care facilities (UNICEF, 2009); our findings complement this work by highlighting the importance of ASHAs' support services in shaping women's perceptions of and desire to deliver in an institutional delivery care facility.

Apart from the aforementioned factors, we found that parity has major role in the use of obstetric care, consistent with studies from Sudan and Ethiopia (Mustafa and Mukhtar, 2015, Wilunda et al., 2015). The decision to seek care is influenced not only by expectant mothers, but also by family members and neighbors. A study in Nicaragua identified the partner support, previous maternal healthcare experiences, and the degree of communication with other women and health workers (Lubbock and Stephenson, 2008) while another study in India highlighted the importance of social capital operated at the community level as a factor influencing the decision to seek care (Story, 2014).

Another important finding from our study is that most people believe that an institutional delivery care facility is necessary only in the case of birth complications. This often resulted in waiting until the last moments of childbirth to seek help from an institutional delivery care facility, leading to delay/non-availability of transportation services and an inability to reach to an institutional delivery care facility in time. A study in Chiapas, Mexico found that the lay population may deem a pregnancy problematic if it i) occurs in a woman with sub-optimal pre-pregnancy conditions (e.g., high or low maternal age, parity (first birth) history of spontaneous abortion), ii) is accompanied by abnormal symptoms in the woman or baby, iii) occurs in unfavorable social circumstances, or iv) is deemed problematic by the woman and/or influential relatives (Tinoco-Ojanguren et al., 2008). We found that a birth was deemed problematic in the sub-optimal pre-pregnancy conditions such as young age, parity, and miscarriage in previous pregnancy. In addition, we found that some pregnancies are deemed to be problematic when nearing the delivery date and time, even in the absence of an adverse pre-pregnancy condition. This was partly because of the psychological pressure of delivery, discomfort felt by the expectant mother, and the delay in delivery after the onset of pain. This also highlights the fact that creating better infrastructure alone, such as roads, vehicles, and institutional delivery care facilities, would be insufficient to promote accessing of and utilization of an institutional delivery care facility. There is also a need to generate awareness among all stakeholders (including pregnant women and their families) of delivery care on the importance of timely decision-making instead of waiting until the last moment.

### 4.3 Limitations

Our study has several limitations. First, this study was based mainly on the demand side perspective, while the perspectives of the health system stakeholders were lacking. Second, we interviewed only one family member per household. Next, though we aimed to collect data from roughly an equal number of the JSY users and non-users, and also with institutional versus home deliveries, we could not identify sufficient number of JSY users with home delivery. Third, we anticipate that some socio-cultural norms relevant to Indian settings, including the stigma associated with accessing the benefits of programs meant for poor people, might have influenced in the under-statement by the participants of the importance of the cash incentive component in the decision to access institutional delivery care. Finally, as with all retrospective data, it is possible that the time since interview may have shaped both the accuracy and content of women's reported birth experiences and knowledge of the JSY.

### 4.4 Policy implications

Our findings have important policy implications, particularly as universal health coverage is now receiving substantial national and worldwide attention (Mills et al., 2012, WHO, 2010b). As there is no single best path for universal coverage, the debate continues on the best mix of healthcare alternatives, including strengthening the supply side of public health system, and the demand side mechanisms such as health insurance and conditional cash-transfer (Kutzin, 2012, WHO, 2010b).

Over the past few years, several low- and middle-income countries including Mexico, Columbia, Nicaragua, Honduras, Brazil and India have introduced public health programs with cash incentives to increase the use of health services among poor people (Attanasio et al., 2005). Previous studies have argued that conditional cash-transfers increase the use of health services (Gertler, 2000, Lagarde et al., 2009, Maluccio and Flores, 2005, Morris et al., 2004, Rivera et al., 2004), but are inconclusive on improving health outcomes. While some studies reported positive impacts (Gertler, 2000, Rivera et al., 2004), another study reported no considerable impacts (Morris et al., 2004). However, the replicability of conditional cash-transfer programs under more deprived settings is still unclear because they depend on effective primary healthcare and mechanisms to disburse payments (Lagarde et al., 2009).

Cash incentives may not be sufficient in isolation for promoting access to institutional delivery care. Findings here highlight the importance of strengthening community health workers' support services and better institutional delivery care facilities as enabling factors in the accessing of institutional birth care. In this context, several related key factors must be taken into consideration. In particular, i) providing focused and adequate support services, especially to pregnant women belonging to the marginalized population groups (e.g. low income, less-educated and in remote locations), ii) incentivizing accessing healthcare for primiparous women and those with problematic pre-pregnancy conditions, iii) generating awareness of and motivation towards best practices among health workers, and iv) strengthening public primary healthcare infrastructure. For the sake of efficiency, it may be particularly beneficial to target primiparous women; coupled with improvements in infrastructure and the quality of care, as women who have a positive first birth experience may be particularly inclined to use delivery care services for subsequent births. Our findings suggest that mistrust in the quality of institutional birth care and the notion of childbirth as a ‘natural event’ that requires no institutional delivery care are some of the impeding factors of accessing of institutional birth care. In light of this finding, policy interventions may also consider accommodating a wider range of childbirth beliefs and practices by providing home-based skilled birth-attendance services.

Given universal awareness and substantial reach of the program, even in high focus states, the focus of the program may now be shifted to sustaining the gains achieved so far by 1) increasing training of ASHAs and knowledge of communities regarding the right timing for taking women to an institutional delivery care facility; 2) strengthening the availability and accessibility of ‘24 × 7’ facilities to handle the Basic Emergency Obstetric Care Centre and Comprehensive Emergency Obstetric Care Services cases; and 3) ensuring the quality of each institutional delivery by supplementing it with basic newborn care.

### 4.5 Concluding remarks

This study found that, overall, the use of maternal healthcare facilities is largely driven by ASHAs' support services rather than the cash incentive component. ASHAs' support services and efforts to generate awareness of the benefits of facility-based childbirth were major enabling factors in the accessing of delivery care. The JSY cash incentive played a lesser role as an enabling factor because of higher opportunity costs in the use of an institutional delivery care facility over home for childbirth. Trust in the skills of traditional birth-attendants and the notion of childbirth as a ‘natural event’ that requires no institutional delivery care were the most prevalent impeding factors. The belief that an institutional delivery care facility would be needed only in cases of birth complications was also a highly prevalent impeding factor.

Our findings suggest that, in order to promote accessing of delivery care in India and other resource-poor country settings, it is important to employ well-trained community health workers at the grass roots level to identify and assist pregnant women. Adequate and intensive support of community health workers is also needed. Moreover, greater emphasis should be placed on education of women and their families about the value of using institutional delivery care facilities. Finally, strengthening of public primary healthcare facilities is needed to ensure the perception and delivery of high quality and responsive care. In sum, a holistic approach focused on marginalized population groups for the continuum of care from family planning, pregnancy, birth, and after delivery is crucial for ensuring that women access institutional healthcare. Institutional delivery care is an important subset of the broader set of maternal healthcare needs, and facilitating women in accessing institutional delivery care is a vital step in improving MCH outcomes.

## Figures and Tables

**Table 1:** Socioeconomic and health profile of the states of Jharkhand, Madhya Pradesh and Uttar Pradesh.

Indicators	Jharkhand	Madhya Pradesh	Uttar Pradesh
Per capita state domestic product (in 2012–13, at current price, Indian rupee)^i^	44,045	44,989	33,137
Literacy rate (percent) (in 2011)^i^	66.4	69.3	67.7
Female literacy rate (percent) (in 2011)^i^	55.4	59.2	57.2
Population below poverty line (percent) (in 2011–12)^i^	37.0	31.7	29.4
Percent of pregnant women received three or more ante-natal check-ups (in 2012–13)^ii^	60.2	71.7	37.8
Percent of institutional delivery (in 2012–13)^ii^	46.2	82.6	56.7
Percent of children with full immunization (Bacillus Calmette–Guérin (BCG) vaccine, three injections of Diphtheria, Pertussis, Tetanus (DPT), three doses of Polio and measles) (in 2012–13)^ii^	69.9	66.4	52.7
Infant mortality rate per 1000 live births (in 2011)^iii^	39	56	53

**Table 2:** Socio-demographic characteristics and the state and district distribution of study participants.

A) Socio-demographic characteristics of the eligible women, spouses and mothers-in-law				
	Eligible women	Spouses	Mothers-in-law	All sample
**N**	41	44	11	96
**Age (standard deviation)**	29 (7)	33 (5)	56 (8)	34 (10)
**Educational qualifications**				
Illiterate	54%	66%	91%	64%
Primary	37%	26%	9%	29%
High-school	9%	7%	0%	7%
College and University	0%	0%	0%	0%
Place of birth*				
Healthcare facility	61%	58%	54%	59%
Home	39%	42%	46%	41%
Parity*				
Primiparous	34%	37%	32%	36%
Mutliparous	66%	63%	68%	64%
**JSY use**	39%	34%	45%	38%

B) Number of participants							
State	Jharkhand		Madhya Pradesh		Uttar Pradesh		**Total**
District	Ranchi	Chatra	Gwalior	Tikamgarh	Ghaziabad	Hamirpur	
**JSY user: Institutional delivery**							
Eligible women	2	2	3	2	2	2	13
Spouses	2	2	2	2	2	2	12
Mothers-in-law	1	0	0	0	1	1	3
**JSY user: Home delivery**							
Eligible women	0	0	2	1	0	0	3
Spouses	0	0	1	1	1	0	3
Mothers-in-law	0	0	1	0	0	1	2
**JSY non-user: Institutional delivery**							
Eligible women	2	2	2	2	3	1	12
Spouses	2	3	2	2	3	2	14
Mothers-in-law	1	0	0	1	1	0	3
**JSY non-user: Home delivery**							
Eligible women	2	4	2	2	2	1	13
Spouses	2	3	2	2	4	2	15
Mothers-in-law	1	0	0	1	0	1	3
**ASHA**	2	2	2	2	2	2	12
**Total**	17	18	19	18	21	15	108

Notes: i) Eligible women are defined as those who gave birth to child anytime during the period of past one year to six months prior to the date of interview; ii) ‘JSY user: Home delivery’ are those women who gave birth to child at home but received the INR 500 JSY cash incentive; iii) *The values corresponding to ‘place of birth’ and ‘parity’ reported by spouse and mothers-in law are for the ‘eligible women in question’ they represent.

## References

[bib1] Andersen R.M. (1995). Revisiting the behavioral model and access to medical care: does it matter?. J. Health Soc. Behav..

[bib2] Attanasio O., Gómez L.C., Heredia P., Vera-Hernandez M. (2005). The short-term impact of a conditional cash subsidy on child health and nutrition in Colombia. Rep. Summ. Fam..

[bib3] Bhattacharyya S., Srivastava A., Avan B.I., Graham W.J. (2012). Quality care at childbirth in the context of health sector reform program in India: contributing factors, challenges and implementation lesson. Health Syst. Policy Res..

[bib4] Carvalho N., Thacker N., Gupta S.S., Salomon J.A. (2014). More evidence on the impact of India's conditional cash transfer program, Janani Suraksha Yojana: quasi-experimental evaluation of the effects on childhood immunization and other reproductive and child health outcomes. PLoS One.

[bib5] Chaturvedi S., De Costa A., Raven J. (2015). Does the Janani Suraksha Yojana cash transfer programme to promote facility births in India ensure skilled birth attendance? A qualitative study of intrapartum care in Madhya Pradesh. Glob. Health Action.

[bib6] Corbin J., Strauss A. (2008). Basics of Qualitative Research: Techniques and Procedures for Developing Grounded Theory.

[bib7] Gertler P. (2000). Final Report: the Impact of Progesa on Health.

[bib8] Government of Chhattisgarh (2009). Notification of JSY Guidelines.

[bib9] Government of India (2006). JSY: Features and Frequesntly Asked Questions, Ministry of Health and Family Welfare.

[bib10] Government of India (2010). Annual Report to the People on Health.

[bib11] Government of India (2010). Janani Suraksha Yojana: Guidelines for Implementation.

[bib12] Government of India (2013). Estimates of Mortality Indicators. Sample Registration System. http://www.censusindia.gov.in/vital_statistics/SRS_Reports_2013.html.

[bib13] Government of India (2013). Maternal Mortality Ratio Bulletin 2011-13. Vital Statistics, Sample Registration System. http://www.censusindia.gov.in/vital_statistics/mmr_bulletin_2011-13.pdf.

[bib14] Government of India (2014). Annual Reports 2012-13.

[bib15] Government of India (2014). Communitisation: about Accredited Social Health Activist (ASHA). http://nrhm.gov.in/communitisation/asha/about-asha.html.

[bib16] Government of India (2014). Janai Surksha Yojana, National Health Mission, Ministry of Health and Family Welfare. http://nrhm.gov.in/nrhm-components/rmnch-a/maternal-health/janani-suraksha-yojana/background.html.

[bib17] Government of India (2015). Annual Reports 2013-14.

[bib18] Government of India (2015). Update on the ASHA Programme, National Health Mission.

[bib19] Gupta S.K., Pal D.K., Tiwari R., Garg R., Shrivastava A.K., Sarawagi R. (2012). Impact of Janani Suraksha Yojana on institutional delivery rate and maternal morbidity and mortality: an observational study in India. J. Health, Popul. Nutr..

[bib20] Hunt L.M., Glantz N.M., Halperin D.C. (2002). Childbirth care-seeking behavior in Chiapas. Health Care Women Int..

[bib21] International Institute for Population Sciences [IIPS] and Macro International (2010). Report on the District Level Household and Facility Survey Round-3, 2007-08: India.

[bib22] Kaushik A., Mishra P., Kesharwani P., Richa, Hussain M.A. (2010). Awareness about JSY among reproductive age women in a rural area of varanasi. Indian J. Prev. Soc. Med..

[bib23] Kutzin J. (2012). Anything goes on the path to universal health coverage?. No. Bull. World Health Organ..

[bib24] Lagarde M., Haines A., Palmer N. (2009). The impact of conditional cash transfers on health outcomes and use of health services in low and middle income countries. Cochrane Database Syst. Rev..

[bib25] Lim S.S., Dandona L., Hoisington J.A., James S.L., Hogan M.C., Gakidou E. (2010). India's Janani Suraksha Yojana, a conditional cash transfer programme to increase births in health facilities: an impact evaluation. Lancet.

[bib26] Lubbock L.A., Stephenson R.B. (2008). Utilization of maternal health care services in the department of Matagalpa, Nicaragua. Rev. Panam. Salud Publica.

[bib27] Maluccio J., Flores R. (2005). Impact evaluation of a conditional cash transfer program: The Nicaraguan Red de Protección Social: Intl Food Policy Res Inst.

[bib28] Mills A., Ataguba J.E., Akazili J., Borghi J., Garshong B., Makawia S. (2012). Equity in financing and use of health care in Ghana, South Africa, and Tanzania: implications for paths to universal coverage. Lancet.

[bib29] Morris S.S., Olinto P., Flores R., Nilson E.A., Figueiro A.C. (2004). Conditional cash transfers are associated with a small reduction in the rate of weight gain of preschool children in northeast Brazil. J. Nutr..

[bib30] Mustafa M.H., Mukhtar A.M. (2015). Factors associated with antenatal and delivery care in Sudan: analysis of the 2010 Sudan household survey. BMC Health Serv. Res..

[bib31] Office of the Registrar General & Census Commissioner (2016). Annual Health Survey Report- a Report on Core and Vital Health Indicators, Part 1, Ministry of Home Affairs.

[bib32] Panja T.K., Mukhopadhyay D.K., Sinha N., Saren A.B., Sinhababu A., Biswas A.B. (2012). Are institutional deliveries promoted by Janani Suraksha Yojana in a district of West Bengal, India?. Indian J. Public Health.

[bib33] Patel R., Ladusingh L. (2015). Do physical proximity and availability of adequate infrastructure at public health facility increase institutional Delivery? A three level hierarchical model approach. PLoS One.

[bib34] Pathak P.K., Singh A., Subramanian S. (2010). Economic inequalities in maternal health care: prenatal care and skilled birth attendance in India, 1992–2006. PLoS One.

[bib35] Paul V.K. (2010). India: conditional cash transfers for in-facility deliveries. Lancet.

[bib36] Paul V.K., Sachdev H.S., Mavalankar D., Ramachandran P., Sankar M.J., Bhandari N. (2011). Reproductive health, and child health and nutrition in India: meeting the challenge. Lancet.

[bib37] Planning Commission (2011). Evaluation Study of National Rural Health Mission (NRHM) in 7 States, Programme Evaluation Organisation.

[bib38] Rai S., Dasgupta R., Das M., Singh S., Devi R., Arora N. (2011). Determinants of utilization of services under MMJSSA scheme in Jharkhand'Client Perspective': a qualitative study in a low performing state of India. Indian J. Public Health.

[bib39] Randive B., Diwan V., De Costa A. (2013). India's conditional cash transfer programme (the JSY) to promote institutional birth: is there an association between institutional birth proportion and maternal mortality?. PLoS One.

[bib40] Randive B., San Sebastian M., De Costa A., Lindholm L. (2014). Inequalities in institutional delivery uptake and maternal mortality reduction in the context of cash incentive program, Janani Suraksha Yojana: results from nine states in India. Soc. Sci. Med..

[bib41] Rivera J.A., Sotres-Alvarez D., Habicht J.-P., Shamah T., Villalpando S. (2004). Impact of the Mexican program for education, health, and nutrition (Progresa) on rates of growth and anemia in infants and young children: a randomized effectiveness study. JAMA.

[bib42] Save The Children (2010). A Fair Chance at Life: Why Equity Matters for Child Mortality. http://www.savethechildren.org.uk/sites/default/files/docs/A_Fair_Chance_at_Life_1.pdf.

[bib43] Sharma P., Semwal J., Kishore S. (2011). A comparative study of utilization of Janani Suraksha Yojana (maternity benefit scheme) in rural areas and urban slums. Indian J. Community Health.

[bib44] Sidney K., Diwan V., El-Khatib Z., de Costa A. (2012). India's JSY cash transfer program for maternal health: who participates and who doesn't- a report from Ujjain district. Reprod. health.

[bib45] Story W.T. (2014). Social capital and the utilization of maternal and child health services in India: a multilevel analysis. Health Place.

[bib46] Strauss A.L., Corbin J.M. (1998). Basics of Qualitative Research: Techniques and Procedures for Developing Grounded Theory.

[bib47] Tinoco-Ojanguren R., Glantz N.M., Martinez-Hernandez I., Ovando-Meza I. (2008). Risk screening, emergency care, and lay concepts of complications during pregnancy in Chiapas, Mexico. Soc. Sci. Med..

[bib48] UNICEF (2009). Coverage Evaluation Survey (CES-2009).

[bib49] United Nations Population Fund- India (2009). Concurrent Assessment of Janani Suraksha Yojana (JSY) in Selected States: Bihar, Madhya Pradesh, Orissa, Rajasthan, Uttar Pradesh.

[bib50] Vellakkal S., Gupta A., Khan Z., Stuckler D., Reeves A., Ebrahim S. (2016). Has India's national rural health mission reduced inequities in maternal health services? A pre-post repeated cross-sectional study. Health Policy Plan..

[bib51] WHO (2010). The World Health Report - Health Systems Financing: the Path to Universal Coverage.

[bib52] Wilunda C., Quaglio G., Putoto G., Takahashi R., Calia F., Abebe D. (2015). Determinants of utilisation of antenatal care and skilled birth attendant at delivery in South West Shoa Zone, Ethiopia: a cross sectional study. Reprod. Health.

[bib53] Zodpey S., VK P. (2014). State of India's Newborns (SION). Public Health Foundation of India.

